# Cerebrovascular responses to a 90° tilt in healthy neonates

**DOI:** 10.1038/s41390-024-03046-1

**Published:** 2024-01-27

**Authors:** Nhu N. Tran, Jason S. Chwa, Kenneth M. Brady, Matthew Borzage, Mary-Lynn Brecht, Jessica X. Woon, Anna Miner, Carlin A. Merkel, Philippe Friedlich, Bradley S. Peterson, John C. Wood

**Affiliations:** 1 Institute for the Developing Mind, The Saban Research Institute, Children’s Hospital Los Angeles, Los Angeles, CA USA; 2 Fetal and Neonatal Institute, Division of Neonatology, Children’s Hospital Los Angeles, Los Angeles, CA USA; 3 Department of Pediatrics, Keck School of Medicine, University of Southern California, Los Angeles, CA USA; 4 Keck School of Medicine, University of Southern California, Los Angeles, CA USA; 5 Lurie Children’s Hospital of Chicago, Northwestern University Feinberg School of Medicine, Chicago, IL USA; 6 School of Nursing, University of California, Los Angeles, Los Angeles, CA USA; 7 Dornsife College of Letters, Arts and Sciences, University of Southern California, Los Angeles, CA USA; 8 Department of Psychiatry, Keck School of Medicine, University of Southern California, Los Angeles, CA USA; 9 Division of Cardiology, Children’s Hospital Los Angeles, Los Angeles, CA USA

## Abstract

**Background:**

Tilts can induce alterations in cerebral hemodynamics in healthy neonates, but prior studies have only examined systemic parameters or used small tilt angles (<90°). The healthy neonatal population, however, are commonly subjected to large tilt angles (≥90°). We sought to characterize the cerebrovascular response to a 90° tilt in healthy term neonates.

**Methods:**

We performed a secondary descriptive analysis on 44 healthy term neonates. We measured cerebral oxygen saturation (rcSO_2_), oxygen saturation (SpO_2_), heart rate (HR), breathing rate (BR), and cerebral fractional tissue oxygen extraction (cFTOE) over three consecutive 90° tilts. These parameters were measured for 2-min while neonates were in a supine (0°) position and 2-min while tilted to a sitting (90°) position. We measured oscillometric mean blood pressure (MBP) at the start of each tilt.

**Results:**

rcSO_2_ and BR decreased significantly in the sitting position, whereas cFTOE, SpO_2_, and MBP increased significantly in the sitting position. We detected a significant position-by-time interaction for all physiological parameters.

**Conclusion:**

A 90° tilt induces a decline in rcSO_2_ and an increase in cFTOE in healthy term neonates. Understanding the normal cerebrovascular response to a 90° tilt in healthy neonates will help clinicians to recognize abnormal responses in high-risk infant populations.

**Impact:**

- Healthy term neonates (≤14 days old) had decreased cerebral oxygen saturation (~1.1%) and increased cerebral oxygen extraction (~0.01) following a 90° tilt.
- We detected a significant position-by-time interaction with all physiological parameters measured, suggesting the effect of position varied across consecutive tilts.
- No prior study has characterized the cerebral oxygen saturation response to a 90° tilt in healthy term neonates.

## Introduction

Tilting the head and body can induce acute hemodynamic alterations in healthy neonates,^1^ however, the normative cerebrovascular response to a large tilt (≥90°) remains unknown. Previous studies have been limited in scope (e.g., using head-only tilt maneuvers, small tilt angles of <90°, or investigating the effects of a tilt on systemic parameters only such as mean blood pressure (MBP) and heart rate (HR)) and show conflicting results in healthy neonates.^1–11^ For instance, head-only tilts (ranging between 30–80°) in healthy term neonates produced both increased HR and decreased MBP,^1,3,5,7,8,10,12,13^ whereas, other studies found no significant change in HR or slightly increased MBP from head-only tilts (ranging between 30–60°).^2,4,6,8,9,11,14^

Furthermore, a couple of studies found no significant change in cerebral oxygen saturation after ≤20° head-tilts in healthy term neonates.^15,16^ Thus, these smaller maneuvers may not reflect the various positions neonates may experience during early life, such as being lifted, carried, or being placed upright in a car-seat.^17^ Prior studies have investigated the effects of tilting on cerebrovascular control in older healthy infants (˃30 days of life), but this study is primarily interested in the healthy neonatal (≤30 days of life) response.^18,19^ Assessing a healthy neonate’s cerebrovascular response to tilting has clinical relevance, due to the complex physiological changes associated with the transition from intrauterine to extrauterine life and the inherent immaturity of hemodynamic functioning.^20,21^ Healthy term neonates may vary in maturation and autoregulatory capacity, and thus, a better understanding of their normal cerebrovascular response to orthostatic stress may identify vulnerable neonates at risk of cerebrovascular dysfunction.^22–24^ Therefore, we sought to: (1) characterize the cerebrovascular response (defined by cerebral oxygen saturation (rcSO_2_) and cerebral fractional tissue oxygen extraction (cFTOE)) to a 90° tilt in healthy term neonates; (2) examine the physiological responses of systemic parameters (systemic oxygen saturation (SpO_2_), HR, breathing rate (BR), and MBP) to a 90° tilt; (3) assess the cerebrovascular stability index (CSI) for each neonate to quantify the brain’s ability to maintain stable oxygenation under orthostatic stress;^25^ (4) calculate the variance of each of the 2-min rcSO_2_ mean differences (“change scores”) across the three tilts; and (5) test whether CSI and the variance of the change scores were associated with age, as older neonates are presumed to have more mature hemodynamic functioning.^21,22^

## Methods

### Design

We performed a secondary descriptive analysis of an ongoing longitudinal cohort study conducted at The Saban Research Institute of Children’s Hospital Los Angeles (CHLA) between July 2018 and May 2022. The overarching study investigates the mechanisms of brain injury and neurodevelopmental delay in infants with congenital heart disease and healthy controls. In this present study, we characterize the cerebrovascular response to a 90° tilt in healthy term neonates within the first 14 days after birth. We obtained written informed parental consent for all neonates prior to study inclusion. We aligned all procedures with the Helsinki Declaration of 1975, as revised in 2008, and the national guidelines for human experimentation (Good Clinical Practice). Moreover, this study was approved by the Committee on Clinical Investigations of CHLA and AltaMed ethics committee.

### Study sample

We recruited healthy expectant mothers and eligible neonates through recruitment flyers and the AltaMed General Pediatrics newborn clinic located within CHLA, respectively. The inclusion criteria for healthy neonates were as follows: (1) ≥37 weeks gestational age at birth; (2) postnatal age ≤14 days at time of examination; (3) enrolled between July 2018 and November 2022. Exclusion criteria encompassed documented instances of: (1) genetic, congenital, or neurologic disorders; (2) complications during pre-, peri-, and postnatal periods; (3) diagnosis of either intrauterine growth restriction or small for gestational age; (4) previous antibiotic treatment for a known infection; (5) maternal diagnosis of substance use disorder; (6) chorioamnionitis; (7) neonatal or maternal steroid use (within the third trimester). Please refer to Tran et al.^25^ and Tran et al.^26^ for more detail on the study sample.

### Cerebrovascular response and physiological measures

**Fig. 1:**
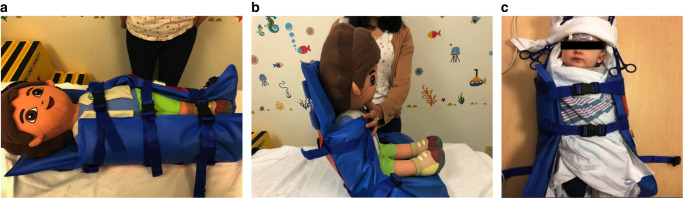
Position changes during the 90° tilt-test. In our 90° tilt-test, neonates were first in a supine (0°) position (**a**) for 2-min and tilted (over 1–2 s) to a sitting (90°) position (**b**) for an additional 2-min. We ensured neonates were not slouched or hunched when being tilted. In the supine position, the heart and brain are of equal height from the ground. In the sitting position, the brain is higher than the heart, resulting in orthostatic stress from gravitational forces. **c** Image of an actual neonatal study participant in the “snuggle-up” during the supine position (permission obtained from the parents).

We used INVOS 5100 C near-infrared spectroscopy (NIRS) (Somanetics, Troy, MI) to measure the cerebrovascular response through rcSO_2_. We placed a neonatal rcSO_2_ sensor on the forehead, a pre-ductal pulse oximetry sensor on the palm of the right hand, 3-lead electrocardiogram on the chest, and an appropriately sized blood pressure cuff around the left brachium. We bundled each neonate in a “snuggle-up” and secured the head to minimize movement. We used the Philips Intellivue MP70 monitors to measure SpO_2_, HR, BR, and MBP. We connected these monitors to the Bernoulli data acquisition system (Cardiopulmonary Corporation, Milford, CT), which sampled all parameters every 5 s. We measured oscillometric MBP at the beginning of each position change. We ensured neonates were asleep and that physiological waveforms were stable prior to testing to minimize artifacts. Notably, we verified a stable electrocardiogram waveform (i.e., visible, regular P and QRS waveforms for HR) and a stable peak and valley waveforms for SpO_2_ that corresponded with the HR and pulse. We also waited at least 5 min after placing the sensors before testing to ensure potential infant stress from the sensor placement did not impact our characterization of the cerebrovascular response. Lastly, we established steady rcSO_2_ values (with no greater than a 5-point change in the numbers) for at least 5 min prior to data collection. We measured all physiological parameters for 2 min while neonates were in a supine (0°) position and for an additional 2-min when tilted (which took 1–2 s) to a sitting (90°) position, over three consecutive tilts. We verified that neonates were not slouching or hunched when tilted to a sitting position, with one hand preserving the vertical position of the back and spine and the other hand maintaining erectness of the head and neck. State changes were recorded if they occurred during the tilt. Our assessment of state changes was subjective, but the following procedures were taken to ensure that neonates had a high likelihood of being asleep: (1) neonates were fed prior to collection of physiologic measurements, (2) sufficient “pre-testing” time (at least 5 min before baseline measures began) was allocated to ensure neonates fell asleep, (3) we assessed changes both visualizing the neonates and on the physiologic monitor (e.g., rate and depth of respirations), and (4) we did not begin baseline measurements until the two study members and the caregiver/parents agreed that the neonate was asleep. Finally, team members were assigned distinct roles to minimize potential errors in data collection, with one person responsible for executing the tilt, while the second person recorded data onto a standardized procedure form. Furthermore, all physiologic parameters were recorded digitally into a data aggregation system that collected all of the values every 5 s in real-time which was downloaded after completion of the study visit for analysis. Please refer to Tran et al.^25^ and Tran et al.^26^ for further detail regarding data collection. Photographs of the position changes from tilting are shown in Fig. 1.

cFTOE was calculated at each five-second interval using rcSO_2_ and SpO_2_ with the equation below.

$${cFTOE}=\frac{({Sp}{O}_{2}-{rcS}{O}_{2})}{{Sp}{O}_{2}}$$

cFTOE represents the balance between oxygen supply and demand in neonatal brains.^27–29^ cFTOE allows for the measurement of the ability of healthy neonates to adapt to procedures that may modify the supply of oxygen, such as tilting. Greater cFTOE indicates increased oxygen consumption by cerebral tissue.

### Cerebrovascular stability index (CSI)

CSI was previously defined in Tran et al.^25^ Briefly, CSI refers to the brain’s ability to maintain stable tissue oxygenation in response to a tilt. Our definition of CSI was based on previous studies in pediatric and adult participants with abnormal cerebrovascular responses to orthostatic stress who demonstrated a more significant reduction in rcSO_2_ compared to controls following positional changes to a standing/upright position.^30–32^ We calculated CSI by subtracting the 2-min rcSO_2_ means of the supine position from the sitting position and averaging the values over three tilts, as shown in the equation below.

$$x={mean}\,{sitting}\,{rcS}{O}_{2}\,{for}\,{tilt}1-{mean}\,{supine}\,{rcS}{O}_{2}\,{for}\,{tilt}1$$

$$y={mean}\,{sitting}\,{rcS}{O}_{2}\,{for}\,{tilt}2-{mean}\,{supine}\,{rcS}{O}_{2}\,{for}\,{tilt}2$$

$$z={mean}\,{sitting}\,{rcS}{O}_{2}\,{for}\,{tilt}3-{mean}\,{supine}\,{rcS}{O}_{2}\,{for}\,{tilt}3$$

$${CSI}=\frac{\left(x+y+z\right)}{3}$$

A CSI close to or equal to zero in healthy infants indicates little change in cerebral oxygen saturation under orthostatic stress, whereas a more negative CSI suggests immature cerebrovascular hemodynamics.

### Cerebral oxygen saturation time series

**Fig. 2:**
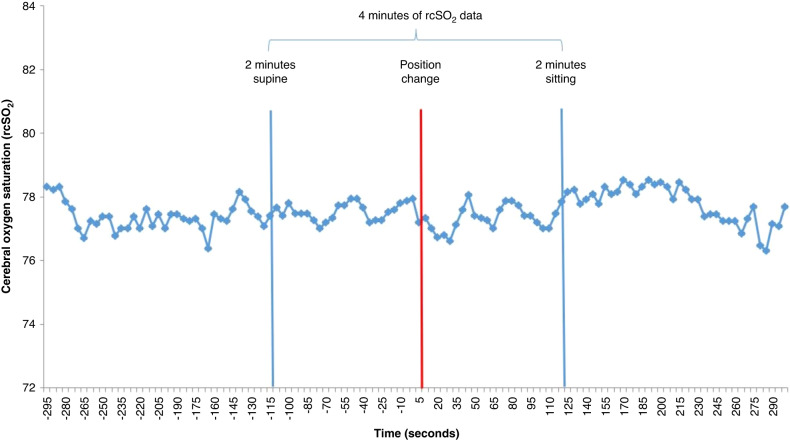
Aggregated rcSO_2_ time series of healthy term neonates. rcSO_2_ was measured at 5-s intervals during a 5-min period in both the supine and sitting positions for all 44 healthy term neonates in our sample. The data from each participant were then combined and aggregated into the above time series. We empirically verified our selection of a 2-min window to capture the cerebrovascular response to a 90° tilt through visual examination of all individual time series, including the above figure. Our aim in selecting a 2-min window was to accurately capture tilt-induced changes while removing the return to baseline.

We demonstrated previously that measuring rcSO_2_ for 2 min in the supine position prior to tilting and then for 2 min in a sitting position following the tilt was a suitable time frame in Tran et al.^26^ As a standard component of our tilt-testing protocol, we assessed rcSO_2_ for 5-min in the supine position and 5-min in the sitting position for the first tilt. For the second and third tilts, we measured rcSO_2_ for 2-min in the supine position and 2-min in the sitting position. We examined the full 10-min of rcSO_2_ data from the first tilt from our entire sample to empirically verify that a 2-min window would provide adequate time for cerebrovascular response to occur and be appropriate for our present analysis (Fig. 2). Additionally, cerebral autoregulation in piglets, modeled as a Butterworth high pass filter, demonstrated that a complete autoregulatory response occurred after 60-s.^33^ While we did not assess cerebral autoregulation in the present study (due to our inability to measure continuous, beat-by-beat blood pressure and correlate it with rcSO_2_), we utilized this 2-min window to guide our characterization of the cerebrovascular response to a 90° tilt. This 2-min timeframe may not have been ideal for the other physiological parameters measured in the study (e.g., MBP), but the primary focus of this study was on cerebral oxygenation and its response to tilting.

### Statistical analyses

We used IBM SPSS Statistics version 28 (IBM Corp., Armonk, NY) to conduct all statistical analyses. We considered two-sided *p* ≤ 0.05 to be statistically significant. We calculated mean values for rcSO_2_, SpO_2_, HR, MBP, BR, and cFTOE for each 2-min segment spent in either the supine or sitting position for each of the three consecutive tilts. We summarized the continuous data as means with standard deviations (±SD) and categorical as frequencies with percentages, respectively.

We investigated the change of each physiological parameter with position, using a mixed-model analysis to account for the repeated-measures of values over time. The dependent variables were rcSO_2_, SpO_2_, HR, MBP, BR, and cFTOE; the independent variables were position and time; the covariates were sex, ethnicity, postconceptional age, and state. The main effect of position determined whether a 90° tilt induced a significant change in each physiological parameter. The position-by-time interaction examined whether the effect of a 90° tilt would vary across consecutive tilts. Lastly, we calculated the variance of the three change scores for each neonate by subtracting the 2-min rcSO_2_ means of the supine position from the sitting position for each tilt:

$${Variance}=\frac{{\left({{{{\rm{x}}}}}-{CSI}\right)}^{2}+{\left(y-{CSI}\right)}^{2}+{\left(z-{CSI}\right)}^{2}}{2}$$

We then used the Shapiro–Wilk test to evaluate normality of CSI, variance, postnatal age, birth gestational age, and postconceptional age. Pearson’s correlation coefficient (for normally distributed data) or Kendall’s τ coefficient (for non-normally distributed data) examined bivariate correlations of CSI and variance with postnatal age, birth gestational age, and postconceptional age.

## Results

**Fig. 3:**
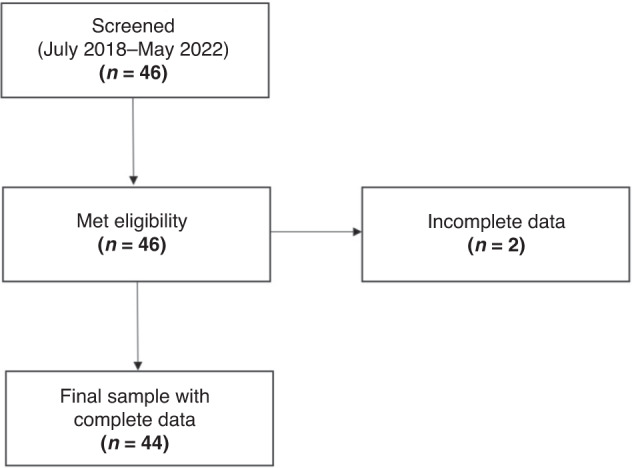
Study inclusion flow chart. Forty-six neonates were screened for eligibility from July 2018 to May 2022. All 46 met study eligibility and underwent testing. Two neonates were excluded from the present analysis as three consecutive tilts were not conducted.

Forty-four neonates were included in the sample (Fig. 3). The median postnatal age was 9.5 (95% CI: 8.0, 12.0) days, birth gestational age was 39.3 (95% CI: 38.6, 39.5) weeks, and postconceptional age was 40.4 (95% CI: 40.1, 41.0) weeks (Table 1). Males comprised 52.3% of the neonates and 65.9% were Hispanic/Latino. The mean values of the physiological parameters in the supine position were: rcSO_2_ = 80.0% (±6.3), SpO_2_ = 97.3% (±1.7), HR = 132.7 (±12.6) bpm (beats per minute), MBP = 61.4 (±11.8) mmHg (millimeters of mercury), BR = 35.8 (±5.7) BPM (breaths per minute), and cFTOE = 0.18 (±0.07) (Table 2). The mean values of the physiological parameters in the sitting position were: rcSO_2_ = 78.9% (±6.1), SpO_2_ = 97.4% (±1.5), HR = 133.7 (±13.2) bpm, MBP = 62.6 (±11.5) mmHg, BR = 33.2 (±4.2) BPM, and cFTOE = 0.19 (±0.07). The mean CSI was −1.1 (±2.7).

**Table 1:** Neonatal demographics.

	Median	95% CI	*N*
Sex			
Male			23 (52.3%)
Female			21 (47.7%)
Ethnicity			
Hispanic/Latino			32 (72.7%)
Caucasian			7 (15.9%)
Asian/Pacific Islander			1 (2.3%)
Other			4 (9.1%)
Postnatal Age (days)	9.5	(8.0, 12.0)	44
Gestational Age at Birth (weeks)	39.3	(38.6, 39.5)	44
Postconceptional Age (weeks)	40.4	(40.1, 41.0)	44

95% CI = 95% Confidence Interval of the Median. Birth Gest. Age = Gestational Age at Birth; Ethnicity-Other = African American, Mixed, Unknown, Other.

**Table 2:** Repeated-measures analysis of the effects of position and the position-by-time interaction on physiological parameters.

rcSO2 (%)	Mean	±SD	β	SE	z	95% CI	*p*
Position							
Supine	80.0	6.3	Ref	-	-	-	**-**
Sitting	78.9	6.1	−0.75	0.15	−5.12	(−1.04, −0.47)	<0.001*
Time	*(Supine/Sitting)*	*(Supine/Sitting)*					
1	(79.8, 79.3)	(0.9, 0.9)	Ref	-	-	-	-
2	(80.1, 78.5)	(0.9, 0.9)	0.18	0.15	1.19	(−0.11, 0.47)	0.23
3	(80.2, 78.9)	(0.9, 0.9)	0.16	0.15	1.05	(−0.13, 0.45)	0.29
Position-by-Time	-	-	-	-	-	-	<0.001*
SpO2 (%)							
Position							
Supine	97.3	1.7	Ref	-	-	-	**-**
Sitting	97.4	1.5	0.31	0.06	4.77	(0.18, 0.44)	<0.001*
Time	*(Supine/Sitting)*	*(Supine/Sitting)*					
1	(97.3, 97.7)	(0.2, 0.2)	Ref	-	-	-	-
2	(97.5, 97.3)	(0.2, 0.2)	0.1	0.07	1.55	(−0.03, 0.23)	0.12
3	(97.2, 97.0)	(0.2, 0.2)	−0.33	0.07	−5.08	(−0.46, −0.21)	<0.001*
Position-by-Time	-	-	-	-	-	-	<0.001*
HR (bpm)							
Position							
Supine	132.7	12.6	Ref	-	-	-	**-**
Sitting	133.7	13.2	0.16	0.47	0.35	(−0.75, 1.08)	0.73
Time	*(Supine/Sitting)*	*(Supine/Sitting)*					
1	(132.8, 133.2)	(1.9, 1.9)	Ref	-	-	-	-
2	(132.6, 134.4)	(1.9, 1.9)	−0.46	0.47	-0.97	(−1.38, 0.47)	0.33
3	(132.6, 133.4)	(1.9, 1.9)	−0.13	0.47	-0.29	(−1.06, 0.79)	0.78
Position-by-Time	-	-	-	-	-	-	0.002*
MBP (mmHg)							
Position							
Supine	61.4	11.8	Ref	-	-	-	**-**
Sitting	62.6	11.5	4.88	0.37	13.24	(4.15, 5.60)	<0.001*
Time	*(Supine/Sitting)*	*(Supine/Sitting)*					
1	(58.9, 63.4)	(1.7, 1.7)	Ref	-	-	-	-
2	(63.5, 63.1)	(1.7, 1.7)	5.25	0.37	14.14	(4.52, 5.98)	<0.001*
3	(61.7, 61.1)	(1.7, 1.7)	3.53	0.37	9.47	(2.79, 4.26)	<0.001*
Position-by-Time	-	-	-	-	-	-	<0.001*
BR (BPM)							
Position							
Supine	35.8	5.7	Ref	-	-	-	**-**
Sitting	33.2	4.2	−3.59	0.4	−8.94	(−4.37, −2.80)	<0.001*
Time	*(Supine/Sitting)*	*(Supine/Sitting)*					
1	(35.6, 32.4)	(0.7, 0.7)	Ref	-	-	-	-
2	(36.7, 33.3)	(0.7, 0.7)	1.11	0.4	2.75	(0.32, 1.90)	0.006*
3	(35.2, 33.9)	(0.7, 0.7)	−0.74	0.41	−1.81	(−1.53, 0.60)	0.07
Position-by-Time	-	-	-	-	-	-	<0.001*
cFTOE							
Position							
Supine	0.18	0.07	Ref	-	-	-	**-**
Sitting	0.19	0.07	0.01	0.002	6.65	(0.007, 0.014)	<0.001*
Time	(Supine/Sitting)	(Supine/Sitting)					
1	(0.18, 0.19)	(0.01, 0.01)	Ref	-	-	-	-
2	(0.18, 0.19)	(0.01, 0.01)	−0.0003	0.002	−0.19	(−0.003, 0.003)	0.85
3	(0.17, 0.19)	(0.01, 0.01)	−0.004	0.002	−2.4	(−0.007, −0.001)	0.016*
Position-by-Time	-	-	-	-	-	-	0.003*

Mixed-model repeated-measures analysis determined the effects of position (supine, sitting), time (1, 2, 3), and the position-by-time interaction on each physiological parameter. Covariates in the model were sex, ethnicity, postconceptional age, and sleep-state. rcSO_2_ = regional cerebral oxygen saturation; SpO_2_ = arterial peripheral oxygen saturation. If significance was achieved under position, parameters were significantly different from the supine position. If significance was achieved under time, parameters were significantly different from the first tilt. If significance was achieved under position-by-time, there was a significant position-by-time interaction on the parameters.

*HR* heart rate, *MBP* mean blood pressure, *BR* breathing rate, *cFTOE* cerebral fractional tissue oxygen extraction.

*2-sided *p* ≤ 0.05.

We detected a significant main effect of position on rcSO_2_ (β = −0.75; 95% CI = −1.04, −0.47; *p* < 0.001), SpO_2_ (β = 0.31; 95% CI = 0.18,0.44; *p* < 0.001), MBP (β = 4.88; 95% CI = 4.15,5.60; *p* < 0.001), BR (β = −3.59; 95% CI = −4.37, −2.80; *p* < 0.001), and cFTOE (β = 0.01; 95% CI = 0.007,0.014; *p* < 0.001) (Table 2). Thus, rcSO_2_ and BR decreased, while SpO_2_, MBP, and cFTOE increased significantly following a 90° tilt. The main effect of position on HR (β = 0.40; 95% CI = −0.46, 1.26; *p* = 0.360) was not statistically significant.

**Fig. 4:**
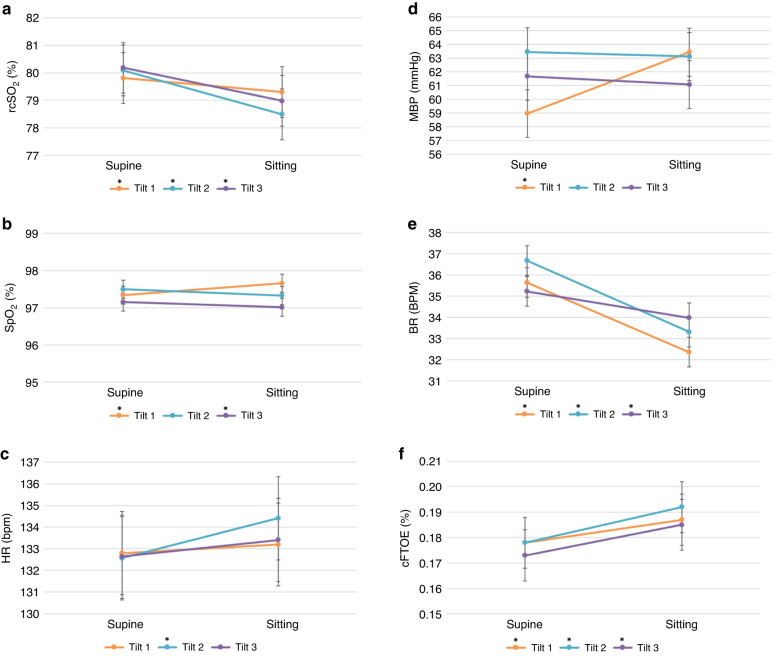
Two-minute supine and sitting marginal means of physiological parameters. Marginal means for each physiological parameter were estimated from the mixed-model repeated-measures analysis shown in Table 2. Error bars denote standard error. rcSO_2_ = regional cerebral oxygen saturation (**a**); SpO_2_ = arterial peripheral oxygen saturation (**b**); HR = heart rate (**c**); MBP = mean blood pressure (**d**); BR = breathing rate (**e**); cFTOE = cerebral fractional tissue oxygen extraction (**f**); bpm = beats per minute; mmHg (millimeters of mercury); BPM = breaths per minute. *2-sided *p* ≤ 0.05.

In addition to the main effect, we also detected significant position-by-time interactions on rcSO_2_ (*p* < 0.001), SpO_2_ (*p* < 0.001), HR (*p* = 0.002), MBP (*p* < 0.001), BR (*p* < 0.001) and cFTOE (*p* = 0.003), indicating that the effects of position on these physiological parameters varied significantly between consecutive tilts. Specifically, rcSO_2_ decreased consistently across the tilts, whereas HR, BR, and cFTOE increased consistently across the tilts. However, MBP was greatly increased from the first tilt but decreased with the second and third tilts. Similarly, SpO_2_ increased slightly from the first tilt but also decreased with the second and third tilts. We depicted the 2-min marginal means of the physiological parameters for each of the three tilts (Fig. 4). We labeled the 2-min means with their respective position (supine, sitting) and time (1, 2, 3) and denoted significant changes with asterisks.

We found no significant correlations between CSI with postnatal age (*r* = 0.06, *p* = 0.57), birth gestational age (*r* = 0.02, *p* = 0.83), and postconceptional age (*r* = 0.05, *p* = 0.66). In addition, we found no significant correlations between the variance of the change scores with postnatal age (*r* = −0.10, *p* = 0.38), birth gestational age (*r* = 0.01, *p* = 0.94), and postconceptional age (*r* = −0.03, *p* = 0.78) (Table 3).

**Table 3:** Bivariate correlations of CSI and variance of change scores with neonatal age characteristics.

	CSI		Variance	
	*r*	*p*	*r*	*p*
Postnatal Age	0.06	0.57	−0.10	0.38
Gestational Age at Birth	0.02	0.83	0.01	0.94
Postconceptional Age	0.05	0.66	−0.03	0.78

Bivariate analyses employed Kendall’s τ coefficient as both CSI and variance were non-normally distributed, as determined by the Shapiro–Wilk test.

*CSI* cerebrovascular stability index, *Variance* variance of 2-min rcSO_2_ mean differences.

*2-sided *p* ≤ 0.05.

## Discussion

This study provides a novel characterization of the healthy neonatal cerebrovascular response to a 90° tilt as we observed significantly reduced rcSO_2_ and increased cFTOE. Additionally, BR decreased whereas SpO_2_ and MBP increased significantly when tilted. Finally, we found the effect of position varied across the three tilts for all physiological parameters (i.e., position-by-time interaction). Neonates are susceptible to changes in the environment, which can turn tilts into potential stressors of themselves, potentially responsible for the changes observed in the monitored parameters. Nevertheless, we also believe the findings observed in the present study can be primarily attributed to the orthostatic challenge associated with a 90° tilt.

Previous studies found conflicting results for the cerebral oxygen saturation response in healthy neonates after a tilt, such as finding no significant change in cerebral oxygenation in after a tilt.^15,16^ This discrepancy with our findings can be attributed to inherent differences in methodology and sample size. Pichler et al.^15^ and Fyfe et al.^16^ conducted 20° and 15° head-tilts in 12 and 17 term neonates, respectively. The orthostatic stress induced by a ≤20° head-tilt may be minimal compared to our 90° whole-body tilt, which may explain the varying results. Similarly, our team previously measured rcSO_2_ in healthy neonates during a 90° tilt, but found no significant difference between the supine and sitting positions.^26^ This difference in results may be due to limited statistical power of the prior study from a smaller sample size (*N* = 16). However, Jarmund et al.^34^ observed a 5–10% reduction in cerebral blood flow velocity (measured via NeoDoppler ultrasound) after a 90° tilt in most of their neonates. Though mean values of all neonates were not calculated, Jarmund et al.’s methods and findings of significantly reduced rcSO_2_ aligned more closely with our results and methodology. Moreover, the average CSI of −1.1 (±2.7) in our sample was similar to the average CSI of −1.2 (±2.8) found in another study with a smaller sample size from a similar cohort.^25^ This further supports the decrease in cerebral oxygenation after a tilt in healthy term neonates.

To the best of our knowledge, no study has examined the effects of postural tilts on cFTOE in healthy term neonates. Previous literature has shown an equally inverse relationship of cerebral oxygen extraction to cerebral perfusion when the cerebral metabolic rate of oxygen is held constant, so higher cFTOE may indicate cerebral hypoxia.^35,36^ Since we accounted for changes in state in our analysis, we controlled for the effects of arousal on the cerebral metabolic rate of oxygen, but a significant increase in cFTOE was observed, nonetheless.^16,35–37^ Thus, the change in cFTOE may be attributed to reduced cerebral perfusion or increased oxygen consumption by brain tissue. Unfortunately, our dataset cannot discern which affected our cFTOE findings. Nevertheless, as rcSO_2_ declined from 80.0% to 78.9% on average and cFTOE increased from 0.18 to 0.19 on average following a 90° tilt, it is unlikely that the reduction in cerebral oxygenation would place healthy neonatal brain at risk of hypoxic injury. However, these findings offer clinicians and investigators an idea of the healthy term neonatal response to large changes in position, which has important implications for managing high-risk infant populations.

Similar to cFTOE, MBP increased significantly in the sitting position. However, the appropriate MBP response to a tilt is unclear in neonates. The majority of studies demonstrated decreased MBP in healthy neonates after head-only tilting (ranging between 30–80°), however, this was tested in much younger (ranging from 2–52 h of age) neonates compared to our present sample.^1,5,12,13^ On the other hand, other studies found increased MBP from slight head-only tilting (from between 30–60°) in much older neonates and infants^8,14^ ranging from 2–3 weeks and 3 months of age. Therefore, it is possible that neonates in our sample had a mature reflex vasoconstrictor response and were able to tolerate the orthostatic stress induced by a large tilt angle (90°) via increased MBP. The increased MBP may also suggest intact cerebral autoregulation in our sample of healthy neonates, since their brain oxygen saturation decreased. Published literature has demonstrated that an inverse relationship between arterial blood pressure and rcSO_2_ demonstrates intact cerebral autoregulation.^38–42^ Nevertheless, accurate assessments of cerebral autoregulation require continuous measurement of arterial blood pressure, which was not conducted in our study.^43^

SpO_2_ increased significantly like MBP, but BR decreased significantly following the 90° tilt. Studies that have investigated SpO_2_ and BR in healthy term neonates utilized 30° head-up tilting.^1,7,44^ Some aligned with our findings, e.g., Thoresen et al.^44^ and Fifer et al.^7^ observed increased SpO_2_ and decreased BR, respectively, following a tilt. However, Chen et al.^1^ found no significant change in SpO_2_ and BR in neonates at 2 h and 24 h of age. Despite both performing 30° head-up tilting, Chen et al. may have had conflicting results with Thoresen et al. as the latter study measured transcutaneous oxygen tension, which can be more sensitive to tilting in comparison to SpO_2_.^1^ Furthermore, unlike Chen et al., we may have observed a significant increase in SpO_2_ due to our use of a 90° whole-body tilt, which would result in a greater increase of the functional residual capacity of the lungs.^45^ The lower BR observed in the sitting position may have been due to the effects of upright positioning on ventilatory-perfusion efficiency of the lungs.^44,46–48^ The depth of respiration may be optimized when in an upright sitting position, thus, adequate oxygenation could be achieved with a lower BR compared to the supine position (where the lungs have reduced pulmonary compliance). Nevertheless, SpO_2_ increased by approximately 0.1% on average in our study, so it is unlikely that this change has clinical relevance, despite achieving statistical significance. The increase in SpO_2_ in our study may have been due to the power to detect changes in our repeated measures mixed model analysis and unrelated to physiological changes.

While HR increased by approximately 0.75% on average within our cohort, this change was not statistically significant. The appropriate HR response to a 90° tilt in healthy term neonates, however, remains unclear. Prior reports have shown that HR increases proportionally to the tilt angle in term neonates.^1,3,5,7–10,13,49^ Yet other studies have observed either no change or a variable change in HR following a tilt.^4,9,11^ These discrepancies can be attributed to inherent differences in methodology and sample composition across studies (i.e., smaller tilt angles (<90°), tilt duration, and postnatal age). Nevertheless, failure to exhibit an increase in HR proportional to the tilt angle may indicate a poorly developed baroreceptor reflex.^4,50^ It is also possible that neonates in our cohort adjusted their stroke volume. Traditionally, it has been presumed that neonates can only modulate cardiac output via adjustments in HR, but more recent studies have nuanced this viewpoint, confirming the substantial role of stroke volume measured via echocardiography.^21,51^ Whether this physiological mechanism remains intact when neonates are subjected to an orthostatic challenge remains unexplored.

We detected a significant position-by-time interaction on rcSO_2_, SpO_2_, HR, MBP, BR, and cFTOE. In other words, the effect of a 90° tilt on each of these physiological measures varied significantly between tilts. rcSO_2_ decreased, however, HR, BR, and cFTOE increased consistently across the 90° tilts. Conversely, MBP increased significantly from the first tilt but decreased slightly in the second and third tilts. Likewise, SpO_2_ increased marginally from the first tilt and decreased with the second and third tilts. Apart from MBP, these small-scale differences may not be clinically relevant. The statistical significance may be due to the statistical power associated with our repeated-measures analysis. For instance, the difference in rcSO_2_ decline across the three tilts was ≤1.5% (0.5%, 1.5%, 1.2%). Nevertheless, no study to date has investigated the position-by-time interaction on cerebrovascular measures when conducting repeated tilts in healthy neonates. Thus, these findings provide insight into our understanding of the healthy neonatal responses to a 90° tilt but repeat experiments should be conducted to ensure reproducibility of this position-by-time interaction.

To the best of our knowledge, only Thoresen et al.^5^ investigated a position-by-time interaction in healthy term neonates, finding that the MBP did not significantly increase by time in response to 30–60° head-only tilt between consecutive tilts. This discrepancy with our findings can be attributed to Thoresen et al. holding each position for 5-min, compared to our 2-min, thus providing more time for habituation to the change in position. It is possible that our neonates did not have enough time in-between tilts for MBP to return to baseline levels in our present study. Therefore, we may not have characterized the MBP response to a 90° tilt accurately for the second and third tilts, and so our findings must be interpreted with caution. Nevertheless, the 2-min window provides an ideal timeframe to capture the cerebrovascular response to a 90° tilt in healthy term neonates.^26,33^

No prior study to date has investigated associations between neonatal age with CSI nor with variance of the change scores. We found no significant correlations between postnatal age, birth gestational age, and postconceptional age with CSI nor with variance of the change scores. We presumed older neonates would have more mature hemodynamic functioning and thus would be able to maintain their rcSO_2_ across tilts.^21,22^ Nevertheless, while differences in hemodynamic functioning may be apparent when comparing neonates to older infant populations, we could not discern any clear trends likely due to the narrow age range of our sample. Furthermore, our calculation of variance could only quantify the magnitude of the deviation of data points from the mean, without considering the direction of those changes. Ultimately, future studies are needed to explore whether certain neonatal characteristics can be predictive of CSI (rcSO_2_ stability while under orthostatic stress) and variance of change scores.

### Limitations

The present study contains some notable limitations. First, rcSO_2_ is a measure of cerebral oxygen saturation, not cerebral perfusion. The use of rcSO_2_ as an estimate of cerebral perfusion requires the assumption that the cerebral metabolic rate of oxygen remained constant across positions. We accounted for changes in state during the tilts in our study, thus we believe the cerebral metabolic rate of oxygen remained constant across positions. Second, we captured the physiologic responses to a 90° tilt in the supine position for 2-min and in the sitting position for another 2-min. However, 2-min in the supine position may have been insufficient resting time for MBP to return to baseline values for subsequent tilts. The primary focus of our paper, however, was on the cerebrovascular response to a tilt. Therefore, the 2-min window would capture this cerebrovascular response.^33^ Third, hemoglobin or blood glucose levels may have affected oxygenation, but we did not measure these values. However, given the healthy status of the neonates in our study we did not have any reason to suspect any significant subject-to-subject variation or change from tilting in these values. Furthermore, invasively measuring hemoglobin and blood glucose during tilt-testing would have likely induced pain, stress, and unintended state changes. Similarly, the intermittent measurements of noninvasive BP may have affected cFTOE. However, the FDA has not approved a device that can continuously and non-invasively measure BPs in neonates, thus we could not perform this mildly less disruptive determination of BPs. Fourth, neonates may have recalled the repeated tilts through memory or physiologically conditioning, affecting their responses. However, performing multiple tilts within a short period of time is consistent with prior studies evaluating cerebrovascular responses in neonates,^15,16^ and the physiologic responses of all parameters changed with each tilt. Thus, we do not believe neonates habituated to the tilts. Lastly, our results may have limited generalizability, as most of the healthy neonates were of Latinx descent and from the Los Angeles Metropolitan area.

## Conclusion

Our study described the cerebrovascular response to a 90° tilt in healthy term neonates. Cerebral oxygen saturation declined from 80.0% to 78.9% on average and cerebral oxygen extraction increased from 0.18 to 0.19 on average following a 90° tilt. Understanding the normal cerebrovascular response to a 90° tilt in healthy term neonates may allow clinicians to recognize abnormalities in high-risk infant populations. Further studies investigating the effects of additional forms of orthostatic challenges on cerebral oxygen saturation in healthy term neonates will help to determination the magnitude of instability or impairment in high-risk infants.

## Data Availability

The datasets generated during and/or analyzed during the current study are available from the corresponding author on reasonable request.
